# Extracts from an Article on Popular Dentistry

**Published:** 1877-12

**Authors:** G. F. J. Colburn


### SELECTIONS.

### EXTRACTS FROM AN ARTICLE ON POPULAR DENTISTRY.

PREPARED BY G. F. J. COLBURN, D.D.S., FOR THE METHODIST QUARTERLY REVIEW.

### *Children's Teeth--How to be Treated.*

The same care and attention required for the preservation of the permanent teeth, is necessary for the deciduous teeth. Nature never intended that the teeth of children should be lost or removed by decay, but that they should remain to fulfill their offices until she should hang out her signal for their removal by causing them to become loose, and give way for the permanent ones by the absorption of their roots. If nature had her course, we should seldom witness a case of irregular or deformed teeth or mouth, now so common. The principal reason of this deformity is, that some of the temporary teeth have been removed, on account of pain and decay, before their time, in consequence of which the space that nature had reserved for a permanent tooth becomes so contracted that when it does appear it is crowded from its position, and is either left thus crowded (in which case it is not only unsightly, but tends to destroy the symmetry that nature intended), or a sound tooth has to be sacrificed to make room for it. Scarcely a week passes that the dentist is not called upon to correct some such irregularity. Children have twenty temporary or deciduous teeth, the germs of which, as well as of the permanent, exist in the jaw even previous to birth, and begin making their appearance about the sixth or seventh month, although the time varies in different children. The period of the eruption of these teeth is the most dangerous and troublesome of the child's existence, and every parent would do well to consult a competent dentist, who will, by proper remedies, palliate the disorders incidental to this period. About the second or third year the temporary teeth are complete and are fully developed, and require the same care to preserve them, both for usefulness and beauty, as is exercised toward the permanent set. All parents should be impressed with the importance of this fact, as they value the health, comfort, and beauty of their offspring. Protect the first set of teeth from the spoiler. Rather let the face or hands of your child remain unwashed than allow him to suffer from unclean or decayed teeth. Early initiate the child into the mysteries of the dental toilet by teaching him to use powder and brush, and that it is necessary that the mouth should be clean to eat his morning meal. Have the toothpick (an instrument more requisite at times than the brush for healthy teeth) brought into requisition after eating, so as to remove all particles of food that remain lodged between the teeth. Many a child would be saved from a great amount of suffering, and the parents spared a great amount of trouble, if these rules were obeyed.

### IMPORTANCE OF EARLY ATTENTION TO THE TEETH AS TO PERSONAL APPEARANCE.

About the sixth year, or soon after, four permanent molar, or double teeth, make their appearance. Let every parent remember this. It is generally supposed that these four teeth belong to the first set, and that if they decay and are removed, they will come again. This is a mistaken idea. They are permanent teeth, and if lost, will be lost forever. No teeth that come after the sixth year are ever shed. At twelve years the second set is usually complete, with the exception of the *dens sapientiæ*, or wisdom teeth, which make their appearance from the eighteenth to the twenty-fourth year. During the eruption of the second set, the beauty and character of the child's countenance is completed, and everything depends upon proper care and attention at this time, to see that the teeth come with regularity, and without being crowded. Should this be the case, the parent may expect a finely-formed mouth; and not, as we often see, a rabbit-narrowness of the mouth and projecting chin, etc., contracting the lips and altering the whole expression of the face.

### CONSEQUENCES OF EARLY NEGLECT OF THE TEETH UPON THE VOICE OF ADULTS.

Another very important reason why the teeth should, early in life, receive the utmost care and professional attention, is the effect they exert upon the articulation. The loss of a single tooth affects the utterance, and invariably produces a hissing or lisping sound in articulating certain words containing the dental vowels, such as, *t*, *d*, *s*, *q* and *j*. All public speakers, especially lawyers, clergyman, and others, should, as they value a correct enunciation and articulation, remember that the teeth were placed by nature to form a certain arch for the express purpose of giving force and purity of utterance. The modulation of the voice also is, in a great measure, dependent upon the shape of the mouth and healthy condition of the teeth and their contiguous parts. Dr. Hill, in his valuable and interesting paper on the "Teeth and Voice," says that "the experience and observation of every thinking man may be called to our aid in support of this position; for it cannot have escaped them that many individuals of profound intellect and brilliant parts make but a sorry figure in their fruitless attempts at oratory and elocution. Every one who has had experience in regard to matters of this kind must have been conscious of great disappointment in not realizing his expectations in regard to certain distinguished men with whose writings he has been long familiar. Having fancied to himself that because they could wield a pen so successfully, they must, therefore, be accomplished speakers, and finding himself sadly mistaken, he is at a loss to account for a circumstance so strange and apparently contradictory. But where lies the difficulty? Certainly not on the score of intellect, for their acquirements are demonstrable from their writings; nor is it because they have never enjoyed the advantages of tuition where elocution was taught. What, then, is the obstacle? We answer, it is to be found in the peculiar conformation of the mouth and the wretched condition of the teeth, giving rise to impediments and difficulties which consitute their misfortune, and of which they are most painfully conscious. Let any one visit a dentist's laboratory and view the casts of different mouths, and he will readily see one reason why people have voices and articulation so various and unlike each other. Some casts represent a mouth not unlike a squirrel's—very narrow and contracted, the upper jaw projecting far over the under, giving a squeaking, effeminate intonation to the voice. Such a shaped mouth is incapable of producing perfect language. We have in our possession two such casts, having the appearance of having been pressed together in a vise. The possessors of these mouths never actually talked, but rather squeaked. No amount of learning or talent, or study of elocution, could ever enable the possessor of such a mouth to become an orator. The whole cause of such deformity was owing to the neglect of the parents while the teeth were being shed. Had a dentist been consulted, the crowded condition of the teeth could have been remedied. There were too many for the space to be occupied, so they became irregular pressing each other, and thereby deforming the mouth. The above case of deformity is but one in thousands that could be related. But even allowing that all due care has been exercised to preserve the symmetry of the dental arch, by having at the proper time the teeth removed, so that there is no crowding or malformation, still, unless the teeth are preserved from decay by proper attention to their health, there will be a difficulty of articulation and enunciation.

Dr. Hill relates a case that occurred in his own practice. "The Rev. Mr. S. was deeply afflicted with a diseased tooth, situated on the right side of the upper jaw. He called at our office for relief. We advised extraction, and it was removed. On the following Sabbath, while engaged in the performance of divine service, he became so annoyed by the loss of that tooth, and so difficult was his enunciation, that he was compelled to stop in the midst of his discourse, and to explain the cause of his difficulty to his congregation. And this from the loss of a single tooth." If such a case of inconvenience arises from the loss of a single tooth, what must be the effect where, from neglect, almost all are lost? A clergyman not long since called upon us to have some slight operation performed, who had, by inattention and neglect, allowed tartar to collect and remain around his teeth, so that his breath was not only very offensive, bnt a number of his teeth were lost from this cause, and others were loose. Such was the condition of his mouth that when he spoke in the pulpit a hissing sound was audible throughout the church. The teeth cannot have too much room. If they were a little separated, they would be less liable to decay. Such men as Henry Clay, Daniel Webster, Patrick Henry, and others, had broad, well-formed mouths. It behooves every one, especially public speakers, to seek to remedy, as far as possible, any deformity that may arise from the loss of the teeth. In a great measure this may be effected by artifical substitutes. In this age of Dentistry there is no deformity or loss that cannot be readily remedied and supplied by the competent dental surgeon in such a manner that, after a little practice, the artificial teeth may be said to make up the deficiency occasioned by the loss of the natural, and fulfill, to a wonderful degree, all purpose of mastication, articulation and beauty.

